# CircRNA_103765 acts as a proinflammatory factor via sponging miR-30 family in Crohn’s disease

**DOI:** 10.1038/s41598-020-80663-w

**Published:** 2021-01-12

**Authors:** Yulan Ye, Liping Zhang, Tong Hu, Juan Yin, Lijuan Xu, Zhi Pang, Weichang Chen

**Affiliations:** 1 Department of Gastroenterology, The First Affiliated Hospital of Soochow University, Suzhou, 215008 Jiangsu China; 2 Department of Gastroenterology, The North District of the Affiliated Suzhou Hospital of Nanjing Medical University, Suzhou, 215008 Jiangsu China

**Keywords:** Cell biology, Genetics, Biomarkers, Gastroenterology

## Abstract

Increasing evidence suggests that circular RNAs (circRNAs) play critical roles in various pathophysiological activities. However, the role of circRNAs in inflammatory bowel disease (IBD) remains unclear. Here we report the potential roles of hsa_circRNA_103765 in regulating cell apoptosis induced by TNF-α in Crohn’s disease (CD). We identify that CircRNA_103765 expression was significantly upregulated in peripheral blood mononuclear cells (PBMCs) of patients with active IBD. A positive correlation with TNF-α significantly enhanced circRNA_103765 expression in CD, which was significantly reversed by anti-TNF-α mAb (infliximab) treatment. In vitro experiments showed that TNF-α could induce the expression of circRNA_103765, which was cell apoptosis dependent, while silencing of circRNA_103765 could protect human intestinal epithelial cells (IECs) from TNF-α-induced apoptosis. In addition, circRNA_103765 acted as a molecular sponge to adsorb the miR-30 family and impair the negative regulation of Delta-like ligand 4 (DLL4). Collectively, CircRNA_103765 is a novel important regulator of the pathogenesis of IBD via sponging miR-30 family-mediated DLL4 expression changes. Blockade of circRNA_103765 could serve as a novel approach for the treatment of IBD patients.

## Introduction

Inflammatory bowel disease (IBD), which mainly includes ulcerative colitis (UC) and Crohn’s disease (CD), is a multifactorial autoimmune disease that is characterized by chronic and recurrent digestive tract inflammatory disorder^1^. In mainland China, with the development of the economy, the locals 's life styles are increasingly westernized, such as higher sugar diet and lower fiber diet, intake of more fast food, which bring about rising incidence and prevalence of IBD^2^. Although the etiology of IBD remains unclear, growing evidence has shown that the pathogenesis of CD is a complex interplay of immunology, genetic predisposition, gut microbiota content and environmental risk factors^1,3,4^.

Traditionally, proinflammatory cytokines produced by immune cells are considered to be crucial in the pathogenesis of IBD. They not only recruit the immune cells and perpetuate inflammation but also have a direct impact on intestinal epithelial cells (IECs), leading to disruption of tight junctions^5–7^ and induction of cell apoptosis^8,9^. Among the various cytokines, the significant role of TNF-α has been illustrated by the dramatic improvement in the management of moderate to severe IBD since the development of anti-TNF-α agents such as infliximab (IFX)^10^.

Circular RNAs (circRNAs) are a newly discovered class of endogenous noncoding RNAs that play important roles in the development and progression of a variety of cancers^11^, autoimmune disease^12^, and nervous system disorders^13^, among others. Owing to their distinct circular covalently closed structure, circRNAs are evolutionally conserved and appear highly stable in the cytoplasm. Recent study has proposed a theory called the competing endogenous RNA (ceRNA) hypothesis that lncRNAs and mRNAs could communicate with and modulate each other through competitively sharing miRNA response elements (MREs), which provides a novel mechanism of gene regulation^14^. It has been shown that circRNAs could also act as ceRNAs to sequester away miRNAs from the target genes. For example, Hansen TB et al. first identified that ciRS-7 may serve as a critical factor in the function of neurons by sponging miR-7^15^. CircHIPK3 is considered a prognostic biomarker in colorectal cancer (CRC) by targeting the c-Myb/circHIPK3/miR-7 axis. However, as the roles of most circRNAs in the initiation and progression of IBD remain unclear and further research is still needed.

The Notch signaling pathway plays a critical role in the development of digestive tract diseases. The Notch pathway contains membrane-bound ligands (Delta-like ligand [DLL]1, DLL3, DLL4, Jagged1, or Jagged2) and receptors (Notch1–4). Direct cell-to-cell interactions by binding a ligand to a receptor causes the release of Notch intracellular domain (NICD) for transcriptional induction of Notch target genes, such as HES1^16^. Moreover, previous studies have shown that DLL4 is the direct binding target of microRNA-30 (miR-30) family members^17–19^. The miR-30 family is a significant member of miRNA family, which is composed of five members (miR-30a, miR-30b, miR-30c, miR-30d, and miR-30e). Recent studies have shown that miR-30 family controls intestinal epithelial cell proliferation and differentiation by targeting a broad gene expression program^20^. MiR-30a-5p ameliorates inflammatory responses through MAPK/ERK signaling^21^. MiR-30e contributed to abnormal small intestinal epithelial differentiation by negatively regulating the Dll4/NICD/Hes1 signaling pathway^19^.

We analyzed the expression profile of circRNAs by microarray analysis in peripheral blood mononuclear cells (PBMCs) from CD patients *versus* healthy controls (HCs) and identified 384 significantly dysregulated circRNAs^22^. In this study, we investigated the expression of circRNA_103765 in the PBMCs of IBD patients by quantitative polymerase chain reaction (qPCR). Then, the functions and mechanisms of circRNA_103765 were deeply explored in vitro. We found that circRNA_103765 was significantly increased in IBD patients. Moreover, enhanced circRNA_103765 expression was distinctly reversed after anti-TNF-α mAb treatment. Knockdown of circRNA_103765 could inhibit cell apoptosis, promote proliferation and downregulate the expression of Delta-like ligand 4 (DLL4). In addition, we performed a luciferase reporter assay and confocal visualization of dual fluorophore RNA- fluorescence in situ hybridization (FISH) and observed that circRNA_103765 could sponge miR-30 family members, including miR-30a-5p and miR-30e-5p. Overall, we determined that circRNA_103765 may regulate DLL4 expression by binding to the miR-30 family. Collectively, these findings indicate that circRNA_103765 might exert regulatory functions in IBD and could be a potential target for therapeutic intervention in IBD therapy.

## Results

### CircRNA_103765 is highly increased in PBMCs of patients with IBD

**Figure 1:**
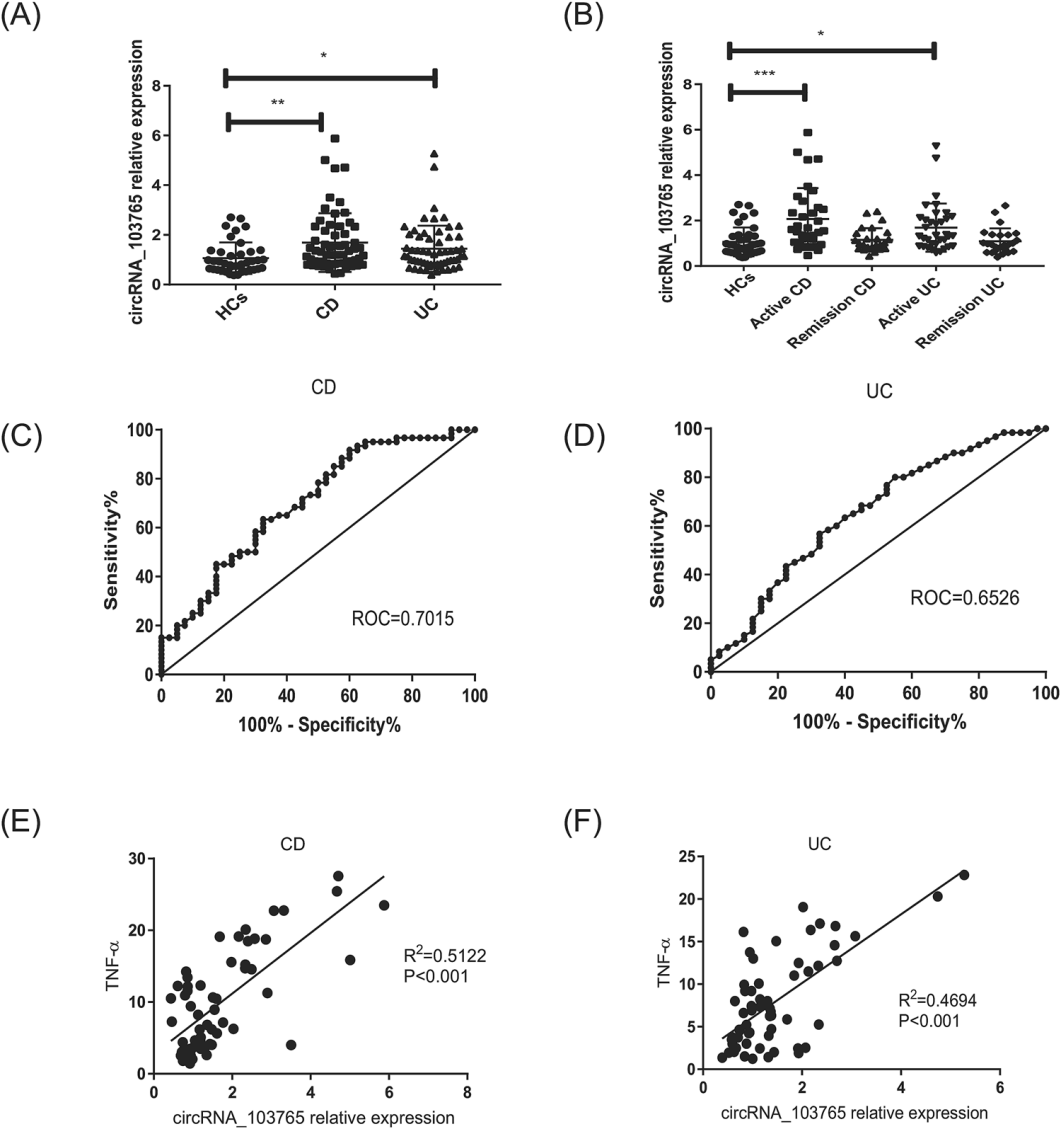
CircRNA_103765 is highly increased in peripheral blood mononuclear cells (PBMC) of patients with IBD. (**A**) qRT-PCR analysis of circRNA_103765 in PBMC from healthy controls (HCs) (n = 40), patients with Crohn’s disease (CD) (n = 60), patients with ulcerative colitis (UC) (n = 60). (**B**) qRT-PCR analysis of circRNA_103765 in PBMC from HC (n = 40), active CD patients (n = 34), CD patients in remission (n = 26), active UC patients (n = 35), UC patients in remission (n = 25). (**C**,**D)** Receiver operating characteristic analysis (ROC) of circRNA_103765 in patients with CD and UC. (**E**,**F**) Correlations of circRNA_103765 expression with TNF­α. *p < 0.05, **p < 0.01, ***p < 0.001.

Our previous study based on a microarray analysis identified 384 dysregulated circRNAs in PBMCs from CD patients compared with HCs. Herein, we focused on circRNA_103765, which is located at chr4:166141085–166184511 and spliced from Kelch-like2 (KLHL2). The kelch-like protein was identified as the genes responsible for inherited diseases, such as a hereditary hypertensive disease-pseudo hypoaldosteronism type II and autosomal dominant retinitis pigmentosa^23,24^. We determined that the expression of circRNA_103765 is upregulated in patients with IBD compared with healthy donors (*P* < 0.05). However, there was no significant difference between the UC and CD groups (Fig. 1A). Moreover, circRNA_103765 expression levels in active CD and UC were distinctly higher than those in remission CD and UC (Fig. 1B).

ROC analysis revealed that circRNA_103765 could differentiate the CD and UC groups from the HCs group (Fig. 1C,D, Supplementary Table S1). The correlations between circRNA_103765 levels and laboratory test results or disease activity of IBD patients are listed in Fig. 1E,F and Supplementary Fig. S1, Supplementary Table S2. We observed a positive correlation of circRNA_103765 with TNF­α (CD: *R*^2^ = 0.5122, *P* < 0.001 and UC: *R*^2^ = 0.4694, *P* < 0.001)**.**

### TNF-α upregulates circRNA_103765 expression in PBMCs of CD patients

**Figure 2:**
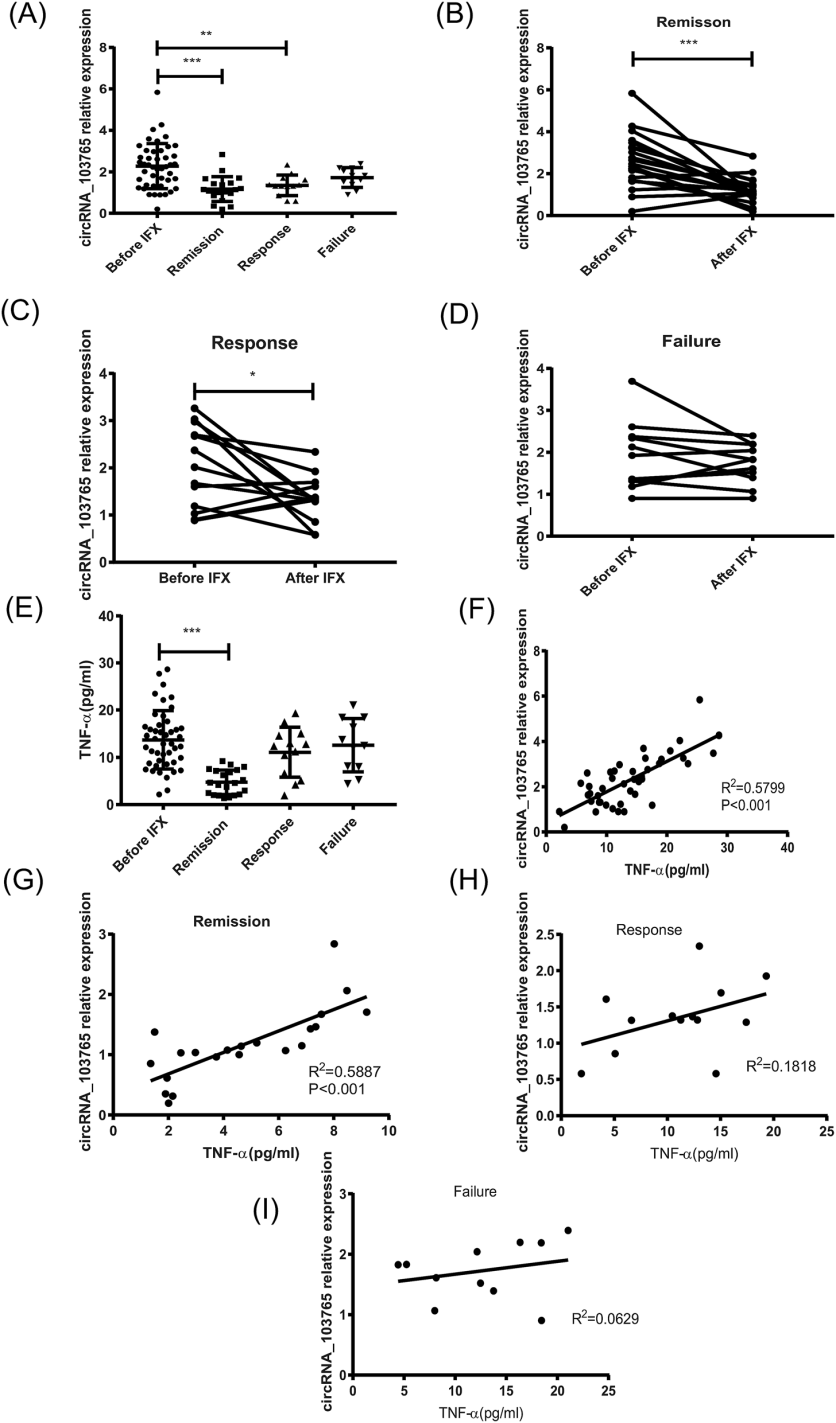
The effect of IFX therapy on circRNA_103765 and TNF­α expression in PBMCs from active CD patients. (**A**) Forty-five active Crohn’s disease (CD) patients received treatment with Infliximab (IFX) as indicated, the changes of circRNA_103765 expression from the remission (n = 21), response (n = 13) and failure (n = 11) groups were analysed. (**B**–**D**) circRNA_103765 expression in PBMCs from 45 active CD patients prior to and after IFX treatment. (**E**) The changes of TNF-α expression from the remission (n = 21), response (n = 13) and failure (n = 11) groups were analysed. (**F**–**I**) Correlations analysis were performed between the levels of TNF-α and circRNA_103765 expression in 45 patients with active CD prior to IFX treatment (**F**) and in clinical remission group (**G**), in the response (**H**) and the failure group (**I**) after IFX treatment (**G**). *p < 0.05, **p < 0.01, ***p < 0.001.

To investigate whether the increased TNF-α in PBMCs contributes to the upregulation of circRNA_103765 in IBD patients, we detected circRNA_103765 expression in PBMCs from CD patients prior to and after treatment with IFX. To this end, 21 patients (46.7%) achieved clinical remission (CDAI < 150), 13 patients (28.8%) did not achieve clinical remission, but they achieved a standard clinical response with a drop in CDAI of ≥ 70 but still with a CDAI ≥ 150. The other 11 participants (24.4%) were unfortunately classified as a failure of IFX due to a CDAI ≥ 150 and a drop in CDAI of ≤ 70 or a changed or increased CDAI from the baseline. Interestingly, we found that circRNA_103765 was considerable decreased in CD patients from the clinical remission and response groups after IFX therapy in comparison with that before IFX induction therapy (Fig. 2A–C). In addition, no change in the expression of circRNA_103765 was observed in the failure group (Fig. 2D).

Moreover, we also observed that the TNF-α level was greatly positively correlated with circRNA_103765 expression in the remission group after IFX therapy in CD patients, as well as before IFX induction therapy (Fig. 2E–G). Nevertheless, no important correlation between circRNA_103765 and TNF-α was found in the response or failure group (Fig. 2H,I). Collectively, these data showed that TNF-α may be able to upregulate circRNA_103765 expression in CD patients and that IFX therapy could markedly reverse the expression of circRNA_103765.

### TNF-α indirectly induces circRNA_103765 transcription by promoting cell apoptosis in a human intestinal epithelial cell line

**Figure 3:**
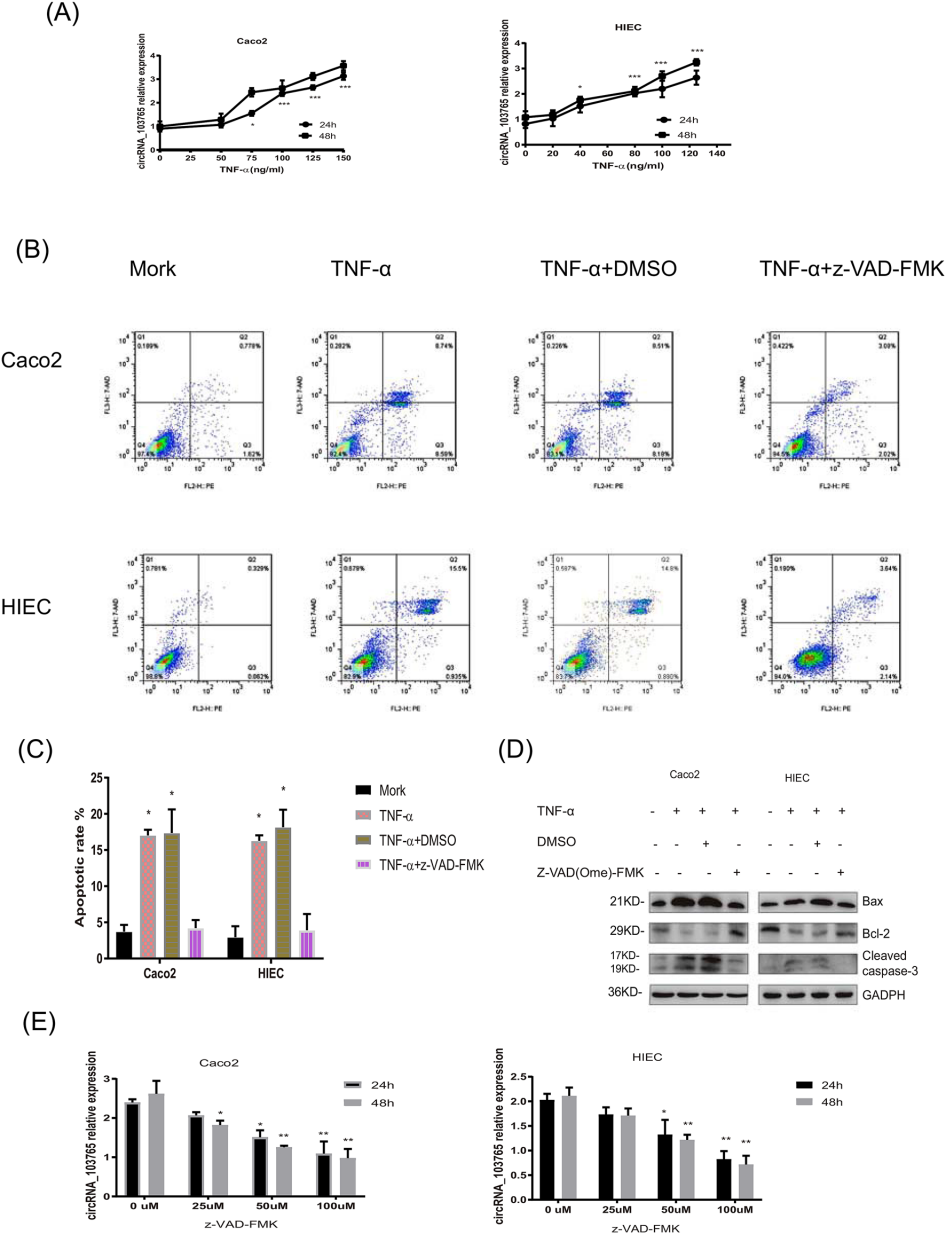
TNF-α induces circRNA_103765 transcription through promoting cell apoptosis in the human intestinal epithelial cell line. (**A**) qRT-PCR analysis of circRNA_103765 expression in Caco2 and HIEC cells treated with TNF-α for 24 h and 48 h. (**B**,**C**) Apoptosis rate was analysed by flow cytometry after treated with TNF-α and z-VAD-FMK. (**D**) The expression levels of apoptosis-related proteins were determined by western blot. (**E**) Effect of various concentrations of z-VAD-FMK on circRNA_103765 expression. Caco2 and HIEC cells were stimulated with TNF-α (100 ng/mL TNF-α for Caco2, 80 ng/mL for HIEC) in the presence of various concentrations of z-VAD-FMK. The level of circRNA_103765 was measured after 24 and 48 h by qRT-PCR. *p < 0.05, **p < 0.01.

To verify the above findings in vitro, we then detected the time course changes in circRNA_103765 expression induced by TNF-α in Caco2 cells and HIECs. As shown in Fig. 3A, TNF-α stimulated circRNA_103765 expression in a dose- and time-dependent manner. The expression level of circRNA_103765 was considerable upregulated in Caco2 and HIECs compared with that in the controls after exposure to TNF-α (*P* < 0.001). Flow cytometry-based Annexin V/PI staining showed that TNF-α significantly increased the apoptotic rate compared with the negative control (*P* < 0.05, Fig. 3B,C). Furthermore, TNF-α increased the levels of the proapoptotic proteins Bax and cleaved caspase-3 and decreased the expression of Bcl-2 (Fig. 3D), which is in line with a previous study^25,26^. Collectively, the data suggest that TNF-α induces circRNA_103765 following IEC apoptosis.

To further determine whether the TNF-α-upregulated circRNA_103765 expression was due to the induction of apoptosis in intestinal epithelial cells, the effect of z-VAD-FMK (a broad-spectrum caspase inhibitor) was determined. Caco2 cells and HIECs were pretreated for 2 h with z-VAD-FMK and then induced with TNF-α. The results showed that z-VAD-FMK induced suppression of circRNA_103765 expression in a concentration- and time-dependent manner (Fig. 3E). The addition of z-VAD-FMK at 25 μM after 24 h had little effect on the expression level of circRNA_103765. However, at 100 μM z-VAD-FMK, the circRNA_103765 level was significantly decreased (*P* < 0.001).The carrier solvent DMSO (< 0.1%) had no effect on circRNA_103765 expression (results not shown). Z-VAD-FMK inhibited the cell apoptotic rate, downregulated Bax and cleaved caspase-3 protein, and increased the expression of Bcl-2, which was induced by TNF-α (Fig. 3B–D).

Taken together, these data suggest that TNF-α-induced circRNA_103765 expression is cell apoptosis dependent.

### Knockdown of circRNA_103765 protects human intestinal epithelial cells from TNF-α-induced apoptosis in vitro

**Figure 4:**
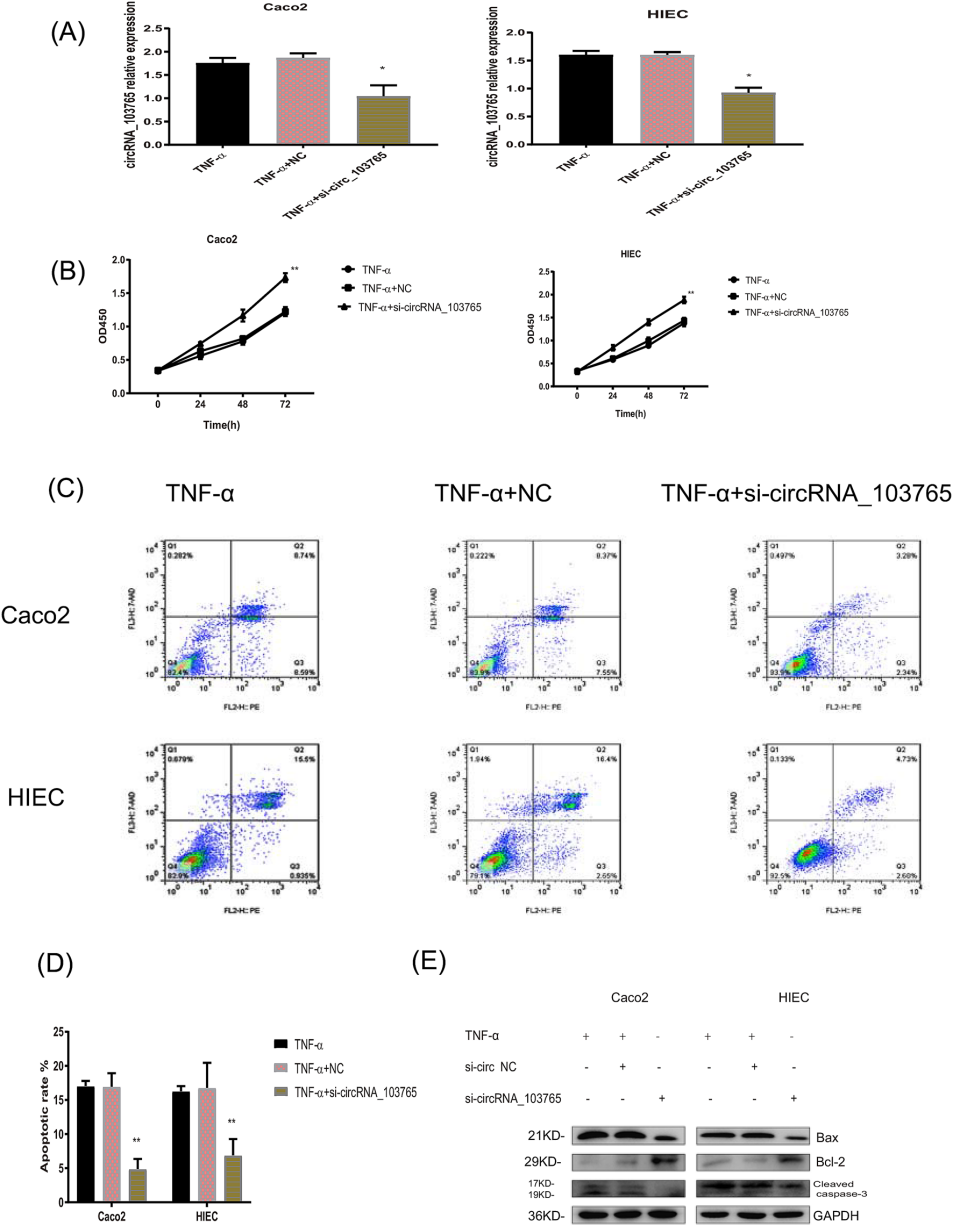
Knockdown of circRNA_103765 protects human intestinal epithelial cells from TNF-α-induced apoptosis. (**A**) Relative expression of circRNA_103765 in TNF-α stimulated Caco2 and HIEC cells treated with negative control siRNA (si-NC) or si- circRNA_103765. (**B**) CCK-8 assay of Caco2 and HIEC cells transfected with negative control siRNA (si-NC) or si-circRNA_103765 at the indicated days. (**C**,**D**) Apoptosis rate were analyzed by flow cytometry after treated with negative control siRNA (si-NC) or si-circRNA_103765. (**E**) The expression levels of apoptosis-related proteins were determined by western blot. *p < 0.05, **p < 0.01.

To explore the biological functions of circRNA_103765 in CD, we synthesized a special siRNA (si-circRNA_103765) targeting the back-splice junction site of circRNA_103765 to knock down circRNA_103765 in a TNF-α-treated Caco2 and HIEC. As illustrated in Fig. 4, the siRNA greatly decreased the expression level of circRNA_103765 (*P* < 0.05, Fig. 4A). Knockdown of circRNA_103765 greatly promoted cell proliferation in both Caco2 cells and HIECs (*P* < 0.05, Fig. 4B). Furthermore, si-circRNA_103765 substantial reduced the apoptotic rate compared with that in TNF-α-induced cells (*P* < 0.05, Fig. 4C,D). Western blot experiments further confirmed this result (Fig. 4E). The above in vitro experiments collectively confirmed that knockdown of circRNA_103765 could protect human intestinal epithelial cells from TNF-α-induced apoptosis, which was consistent with the clinical findings.

### Hsa_circRNA_103765 functions as a sponge for the miR-30 family

**Figure 5:**
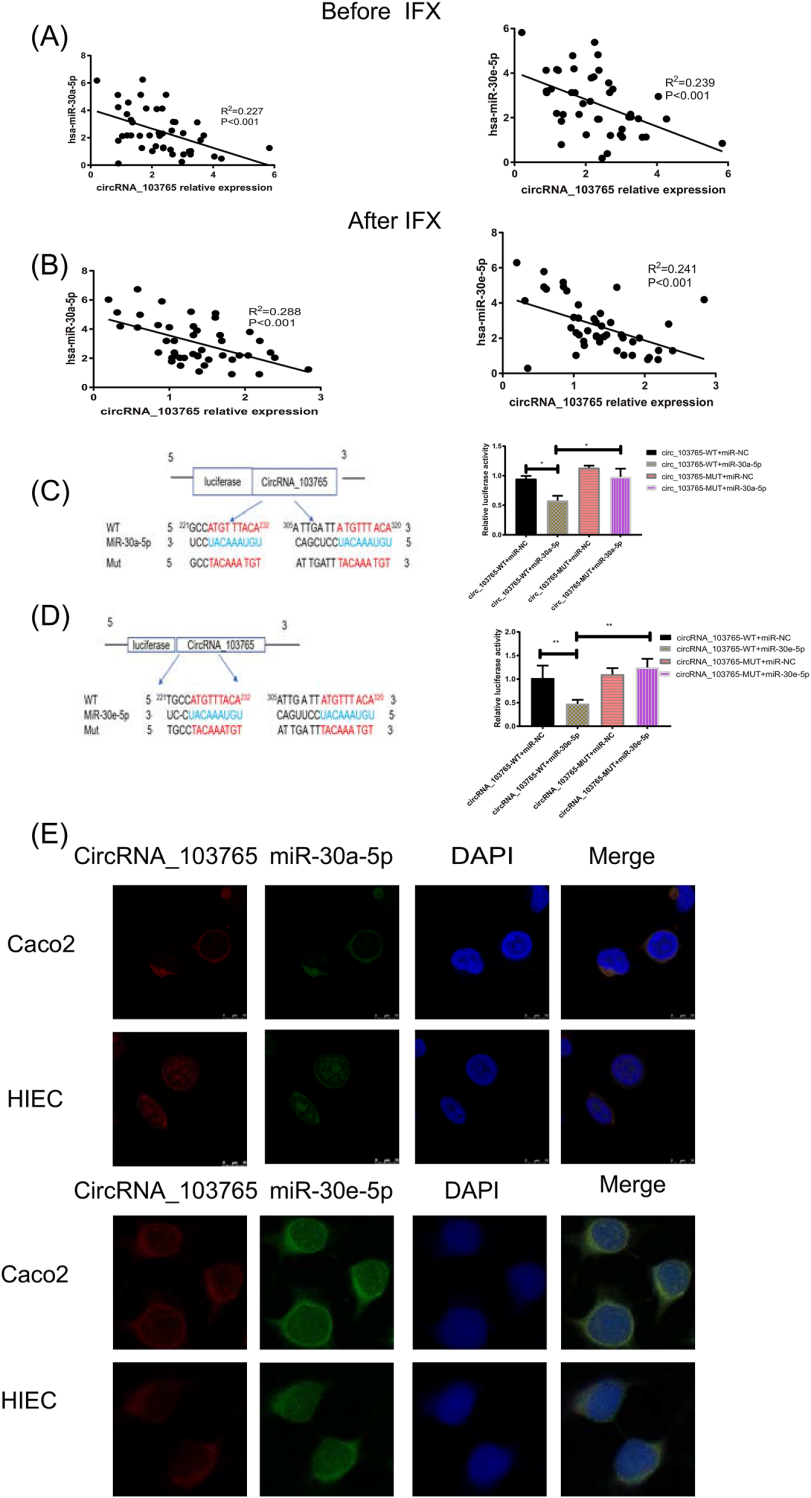
CircRNA_103765 functions as a sponge for miR-30 family. (**A**,**B**) Correlations of circRNA_103765 expression with miR-30a-5p and miR-30e-5p in active CD patients prior to and after IFX treatment (n = 45). (**C**,**D**) Schematic illustration of circRNA_103765-WT and circRNA_103765-Mut luciferase reporter vectors. The relative luciferase activities were detected in 293T cells after transfection with circRNA_103765-WT, circRNA_103765-Mut and miR-30a-5p/miR-30e-5p mimics or miR-NC, respectively. (**E**) FISH was performed to observe the cellular location of circRNA_103765 (red) and miR-30a-5p/miR-30e-5p (green) in cells (magnification, × 40, scale bar, 10 μm). *p < 0.05, **p < 0.01.

To elucidate the molecular mechanism of circRNA_103765, we first predicted the targets of circRNA_103765 according to the TargetScan and miRanda databases. The results showed that circRNA_103765 contains a conserved target site of the miR-30 family (miR-30a-5p, miR-30b-5p, miR-30d-5p, miR-30e-5p) (Supplementary Fig. S2). Thus, we investigated the corresponding miR-30 expression in PBMCs from CD patients prior to and after treatment with IFX. Interestingly, the expression level of circRNA_103765 was negatively correlated with miR-30a-5p and miR-30e-5p expression (*P* < 0.001) (Fig. 5A,B). However, there was no significant correlation between circRNA_103765 and miR-30b-5p or miR-30d-5p (Supplementary Fig. S3). On this basis, a dual luciferase reporter assay, was conducted to validate the sponging relationship between circRNA_103765 and miR-30a-5p and 30e-5p in 293T cells. The results demonstrated that miR-30a-5p and miR-30e-5p mimics could markedly decrease the luciferase activity of the circRNA_103765 WT group but not the mutant group (*P* < 0.05 for miR-30a-5p; *P* < 0.001 for miR-30e-5p, Fig. 5C,D). We performed a FISH assay to observe the subcellular localization of circRNA_103765, miR-30a-5p, and miR-30e-5p. It was found that most circRNA_103765 (red) and miR-30a-5p/30e-5p (green) were mainly colocalized in the cytoplasm (Fig. 5E).

Together, these data demonstrated that circRNA_103765 could act as a sponge of miR-30a and miR-30e in a human intestinal epithelial cell line.

### CircRNA_103765 promotes human intestinal epithelial cell proliferation and apoptosis through the circRNA_103765/miR-30/DLL4 axis

**Figure 6:**
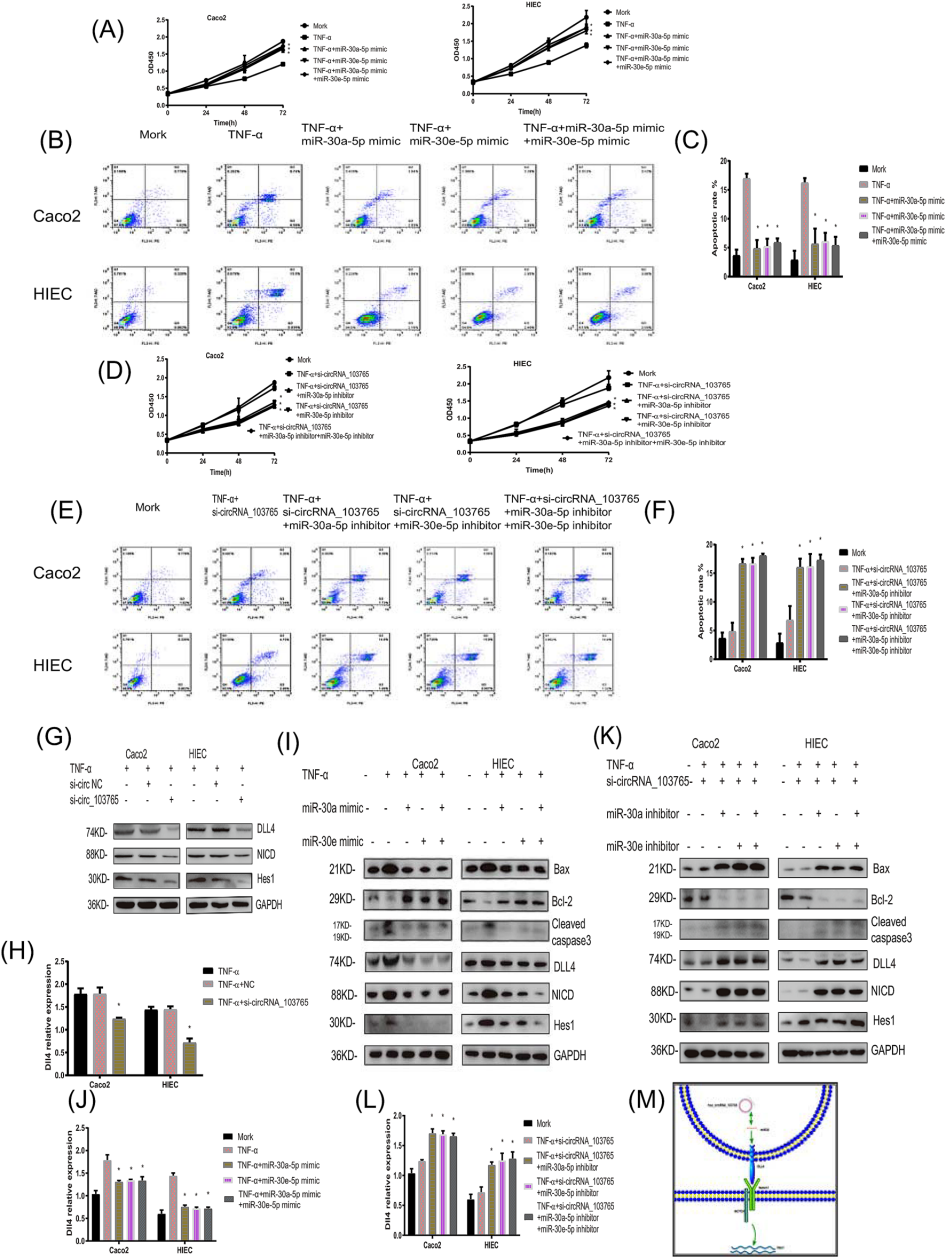
CircRNA_103765 promotes human intestinal epithelial cells proliferation and apoptosis through circRNA_103765/miR-30/DLL4 axis. (**A**,**D**) The cell proliferation was determined after transfection with indicated vectors, mimics or inhibitors at the indicated days. (**B**,**C**,**E**,**F**) Apoptosis rate was analysed by flow cytometry after transfection. (**G**–**L**) Relative expression of DLL4 and downstream cell cycle-related molecules and apoptosis-related molecules at protein level in cells transfected with indicated vectors, mimics or inhibitors was determined by western blot. (**M**) Schematic diagram of how circRNA_103765 sponge miR-30 mediated DLL4 expression. *P < 0.05.

Previous studies have confirmed that DLL4 is the direct binding target of miR-30 family members^17–19^. For further confirmation that circRNA_103765 can act as a competitive endogenous RNA (ceRNA) through the circRNA_103765/miR-30/DLL4 axis, we performed rescue experiments, miR-30a-5p and miR-30e-5p mimics (miR-30a-5p/miR-30e-5p (+)), miR-30a-5p and miR-30e-5p inhibitors (miR-30a-5p/miR-30e-5p (−)) were synthesized and transiently transfected into Caco2 cells and HIECs. As expected, transfection of miR-30a-5p mimics or miR-30e-5p mimics rescued the cell proliferation and apoptosis effects induced by TNF-α (Fig. 6A–C). Conversely, miR-30a-5p inhibitors or miR-30e-5p inhibitors reversed the proliferation and apoptosis effects of circRNA_103765 knockdown in Caco2 cells and HIECs (Fig. 6D–F).

In addition, western blot and qRT-PCR demonstrated that the TNF-α-induced upregulation of circRNA_103765 enhanced the protein and mRNA levels of DLL4 and the downstream proteins NICD and Hes1, while knockdown of circRNA_103765 decreased the levels of these proteins (Fig. 6G,H), and the effects caused by upregulation or silencing of circRNA_103765 could be reversed by miR-30a-5p and miR-30e-5p mimics or inhibitors, respectively. Interestingly, co-expression of miR-30a-5p and miR-30e-5p mimics or inhibitors at an equivalent total concentration did not further increase or decrease DLL4 expression, suggesting that there were no synergistic or additive effects between miR-30a-5p and miR-30e-5p (Fig. 6I–L).

In summary, these data demonstrated that circRNA_103765 might serve as a ceRNA for miR-30a-5p/miR-30e-5p to regulate the expression level of DLL4 (Fig. 6M).

## Discussion

In recent years, a larger number of circRNAs have been discovered in multiple disease and cell lines by high-density microarrays and next-generation sequencing technology. Due to their disease-specific expression, molecular stability and conservatism, circRNAs can regulate various biological processes to act as miRNA sponges, and they are considered ideal diagnostic or therapeutic candidates. To the best of our knowledge, the biological function of most circRNAs remains largely unclear in IBD studies.

Here, our previous research analyzed the expression profile of circRNAs by microarray analysis in CD patients versus healthy controls (HCs) and identified 384 significantly dysregulated circRNAs^22^. Subsequently, we identified a novel circRNA termed circRNA_103765 that was obviously upregulated in active IBD patients and significantly correlated with the level of TNF­α in CD. To further investigate whether increased TNF-α contributes to the upregulation of circRNA_103765 in IBD patients, we detected circRNA_103765 expression in CD patients prior to and after treatment with IFX. Interestingly, we determined that TNF-α could upregulate circRNA_103765 expression in CD patients. Moreover, our data also show that effective anti-TNF-α treatment resulted in a rapid reduction in circRNA_103765 expression, which may be able to ‘predict’ whether treatment with IFX is effective in IBD patients. Furthermore, in vitro cytology experiments also confirmed this result. Moreover, our research further showed that TNF-α-induced circRNA_103765 expression was cell apoptosis dependent, while knockdown of circRNA_103765 could protect human intestinal epithelial cells from TNF-α-induced apoptosis.

Growing evidence has demonstrated that some circRNAs can serve as functional miRNA sponges to regulate the expression of miRNA target genes in various human diseases. In our study, we found that circRNA_103765 contained the MRE of the miR-30 family through bioinformatics analyses. We also found that the expression level of circRNA_103765 was negatively correlated with miR-30a-5p and miR-30e-5p in CD patients prior to and after treatment with IFX. Furthermore, a dual-luciferase reporter assay confirmed that circRNA_103765 could interact with miR-30a-5p and miR-30e-5p directly. FISH assays showed that circRNA_103765 and miR-30a-5p/miR-30e-5p were colocalized in the cytoplasm. Herein, circRNA_103765 could act as a sponge of miR-30a and miR-30e in IBD. Moreover, previous studies have confirmed that DLL4 is the direct binding target of miR-30 family members^17–19^. Notch Delta-like ligand 4 (DLL4) is a well-known transmembrane protein that can activate Notch receptors on adjacent cells. Furthermore, Notch signaling is a key signaling pathway that is involved in cell–cell communication and development^27^. It regulates a variety of cellular processes, such as differentiation, proliferation, apoptosis, and inflammation in IECs^28–30^. In this study, to validate the crosstalk between circRNA_103765 and DLL4, we revealed that the TNF-α-induced upregulation of circRNA_103765 enhanced the protein and mRNA levels of DLL4 and the expression of the downstream proteins NICD and Hes1, while knockdown of circRNA_103765 decreased the levels of these proteins. Moreover, the effects caused by upregulation or silencing of circRNA_103765 could be reversed by miR-30a-5p and miR-30e-5p mimics or inhibitors, respectively, which supports our hypothesis that circRNA_103765 functions as a ceRNA to promote DLL4-mediated cell apoptosis by decoying the miR-30 family in CD.

Significant crosstalk between TNF-α and Notch signaling has been observed. For example, TNF-α activates Notch signal-related proteins in rheumatoid arthritis^31^, but TNF-α suppresses the Notch signaling pathway in skeletal myogenesis^32^. Moreover, Notch ligand Delta-1 reduces TNF-α-induced apoptosis by decreasing the activation of caspases^33^. It seems that there would be certain reciprocal regulation between the two signals, which is cell- and context-dependent. Our newly found evidence indicates that the upregulation of circRNA_103765 associated with TNF-α is under the control of the Notch signaling pathway by sponging the miR-30 family, which is a proinflammatory and proapoptotic factor in IECs. However, how DLL4/Notch signaling interferes with TNF-α signaling during the apoptosis process in IBD requires further study.

In summary, our study indicates that circRNA_103765 is a novel important regulator of the pathogenesis of IBD by regulating the apoptosis of IECs via miR-30 family-mediated DLL4 expression changes. CircRNA_103765 knockdown was able to ameliorate cell apoptosis in vitro, further confirming the above findings. Our study provides new insight into the pathogenic role of circRNA_103765 in IBD and suggests that targeted therapy directly against circRNA_103765 could be a novel approach for the treatment of IBD patients.

Because our experiment is only based on clinical samples and cell lines, further in vivo studies are needed to evaluate its utility. Herein, the newly discovered link between circRNA_103765 and IBD will hopefully provide a better understanding of the disease, with potential for improved diagnosis or therapy in the future.

## Methods

### Patients

IBD patients (60 with CD and 60 with UC) were prospectively enrolled at the Department of Gastroenterology of the First Affiliated Hospital of Soochow University and the North District of the Affiliated Suzhou Hospital of Nanjing Medical University (Jiangsu, China). Forty healthy controls were included. The demographic and clinical information are listed in Table 1. The diagnosis of IBD was based on clinical manifestations, radiological findings, endoscopic examination and histological findings. The severity of diseases was scored according to the CD Activity Index (CDAI) for the diagnosis of patients with CD and the Mayo score for UC. Active IBD patients were defined as CDAI ≥ 150 or Mayo score > 2.

**Table 1:** Clinical characteristics of IBD patients and controls.

Characteristic	CD (A/R)	UC (A/R)	HCs
Number of patients	60 (34/26)	60 (35/25)	40
Mean age (yr)	35.94 ± 10.35	40.33 ± 11.58	38.24 ± 10.85
**Gender**			
Male	37	33	21
female	23	27	19
Range (years)	16–65	25–70	18–65
Disease duration (years)	7.2 ± 5.3	8.4 ± 6.3	
**Disease location: CD, n**			
L1	26		
L2	13		
L3	21		
L4	0		
**Disease behavior: CD, n**			
B1	18		
B2	33		
B3	9		
**Disease location: UC, n**			
E1		25	
E2		22	
E3		13	
CRP (mg/L)	77.06 (14.66–133.14)	47.73 (3.81–113.27)	
TNF-α (pg/mL)	7.74 (3.75–14.68)	6.70 (3.39–11.98)	
CDAI score	182.15 (121.50–300.66)		
Mayo score		1.0 (3.0–5.0)	

The data are presented as the mean ± SD or medians (1/4–3/4 quarters). Patients with isolated upper disease (L4) were excluded. *L1* terminal ileum, *L2* colon, *L3* ileocolon, *B1* non-stricture and non-penetrating, *B2* stricturing, *B3* penetrating, *E1* rectum, *E2* left side, *E3* extensive, *CD* Crohn’s disease, *UC* ulcerative colitis, *HCs* healthy controls, *CRP* C-reactive protein, *TNF-α* tumor necrosis factor α, *CDAI* CD Activity Index, *A/R* active/remission.

PBMCs were promptly isolated after blood sample collection from all subjects according to the manufacturer’s protocol (GE Healthcare) and then frozen at − 80 °C.

### Anti-TNF-α mAb treatment of patients with CD

Forty-five active CD patients were treated with anti-TNF-α monoclonal antibody (infliximab (IFX), 5 mg/kg; Switzerland) at 0, 2 and 6 weeks^34^ (Table 2). Clinical response to IFX was assessed at week 12 after initial infusion. Clinical remission was defined as CDAI < 150, and clinical response was defined as a decrease of 70 or more points in the CDAI score compared with the baseline index. The failure of IFX therapy included patients whose CDAI was not greatly changed or increased^35,36^.

**Table 2:** Clinical characteristics of CD patients with IFX treatment.

Characteristics	Remission group	Response group	Failure group
Number of patients	21	13	11
Mean age (years)	23.6 ± 8.5	27.4 ± 9.4	28.1 ± 8.0
Gender (M/F)	11/10	8/5	6/5
Disease duration (Month)	20.6 ± 11.8	38.7 ± 15.4	62.4 ± 28.3
Disease location (L1/L2/L3/L4)*	7/6/8/0	5/3/5/0	3/3/5/0
Behaviour (B1/B2/B3)*	15/6/0	6/6/1	4/6/1
Perianal disease (yes/no)*	4/17	3/10	1/10
Past IFX exposure (yes/no)	5/16	2/11	2/9
Concomitant azathioprine (yes/no)	9/12	8/5	7/4
CRP (mg/L)	6.32(3.34–10.53)	14.56(7.93–25.53)	15.12(10.42–32.18)
TNF-α (pg/mL)	4.57(2.09–2.26)	12.37(5.85–14.82)	12.48(8.00–1843)
CDAI score	64.63(30.53–143.87)	178.63(158.12–220.63)	201.30(159.01–260.38)

*Montreal Classification. The data are presented as the mean ± SD or medians (1/4–3/4 quarters). Patients with isolated upper disease (L4) were excluded. *L1* terminal ileum, *L2* colon, *L3* ileocolon, *B1* non-stricture and non-penetrating, *B2* stricturing, *B3* penetrating, *E1* rectum, *E2* left side, *E3* extensive, *CD* Crohn’s disease, *UC* ulcerative colitis, *HCs* healthy controls, *CRP* C-reactive protein, *TNF-α* tumor necrosis factor α, *CDAI* CD Activity Index.

### Cell culture and inflammatory model

Human intestinal epithelial cell lines (Caco-2 and HIEC) were purchased from Shanghai Suer Biological Technology Co., Ltd. (Shanghai). The human embryonic kidney 293T (HEK293T) cell line was donated by Dr Chen at the First Affiliated Hospital of Soochow University (Suzhou). The Caco-2 and 293T cell lines were cultured in high-glucose Dulbecco’s modified essential medium (HyClone) containing 10% fetal bovine serum (Gibco) and 1% penicillin and streptomycin (Gibco) in a 5% CO2 atmosphere at 37 °C. HIECs were cultured in RPMI 1640 medium (HyClone) according to the recommended protocols.

TNF-α (MedChemExpress) was added to the medium to mimic an inflammatory background as previously described^37^.

### RNA interference and transfection assay

For transient transfection assays, small interfering RNAs (siRNAs) targeting the back-splice junction sites of circRNA_103765 (si-circRNA_103765) and the non-targeting sequences were designed and synthesized by GenePharma (Shanghai; Table 3). MiR-30 (miR-30a-5p/miR-30e-5p) mimics and inhibitors were purchased from GenePharma (Shanghai). According to the manufacturer’s instructions, cells were transfected using Opti-MEM and Lipofectamine 3000 reagents (Invitrogen)^38,39^.

**Table 3:** Sequences of siRNAs used in this study.

Definition	Sequences
si-circ	5′-CAACACCUAUGCAGAUGCATTUGCAUCUGCAUAGGUGUUGTT-3′
si-NC	Sense 5′-GCGACGAUCUGCCUAAGAU dTdT-3′ Antisense 5′-AUCUUAGGCAGAUCGUCGC dTdT-3′

### Quantitative real-time PCR (qRT-PCR)

Total RNA was isolated with Trizol according to the manufacturer’s protocols (Invitrogen). The PrimeScript Real­Time Reagent kit (Takara) was used for reverse transcription. Afterwards, the TB Green Premix Ex Taq II kit (TaKaRa) was used to quantitate the expression levels of circRNA_103765 and DLL4. Hairpin-it microRNA and U6 snRNA Normalization RT-PCR Quantitation Kit (GenePharma) were used to quantify miR-30a-5p, miR-30b-5p, miR-30d-5p, miR-30e-5p and U6 levels. β-Actin was used as an internal reference for circRNA and mRNA, while U6 was used for miRNA^40^. The specific primers used are indicated in Table 4.

**Table 4:** Sequences of primers used in this study.

Gene	Primer sequences
circRNA_103765	F: 5′-TGCATGTACCGACCTTCTGA-3′
	R: 5′-GCTTCTGATGACCCTGCTTT-3′
DLL4	F: 5′-GTCTCCACGCCGGTATTGG-3′
	R: 5′-CAGGTGAAATTGAAGGGCAGT-3′
β-Actin	F: 5′‑GTGGCCGAGGACTTTGATTG-3′
	R: 5′-CCTGTAACAACGCATCTCATATT-3′
miR-30a-5p	F: 5′-CAGTGCTGTGTAAACATCCTCG -3′
	R: 5′-TATGGTTGTTCACGACTCCTTCAC-3′
miR-30b-5p	F: 5′-GCGCTGTAAACATCCTACAC-3′
	R: 5′-GTGCAGGGTCCGAGGT-3′
miR-30d-5p	F: 5′-GCTGTAAACATCCCCGAC-3′
	R: 5′-GTGCAGGGTCCGAGGT-3′
miR-30e-5p	F: 5′-GCGCTGTAAACATCCTTGAC -3′
	R: 5′-GTGCAGGGTCCGAGGT-3′
U6	F: 5′-CTCGCTTCGGCAGCACA-3′
	R: 5′-AACGCTTCACGAATTTGCGT-3′

*DLL4* delta-like ligand 4.

### Cell viability assay

Cell viability assays were performed by a Cell counting kit-8 (CCK-8 kit) (Dojindo Laboratories) according to the manufacturer’s instructions. Approximately 1 × 10^3^ transfected or treated Caco-2 cells or HIECs were incubated in 96-well plates. The optical density (OD) at 450 nm was measured by a Multimode Spectral Scanning Reader (Thermo Scientific).

### Apoptosis analysis

Apoptosis assays were performed with the Annexin V-fluorescein isothiocyanate (FITC) Apoptosis Detection Kit (BD Biosciences Pharmingen) according to the manufacturer’s protocols. Apoptotic cells were analyzed using a flow cytometer (BD Biosciences), and the data were analyzed using FlowJo 7.6 Software^38^. The most widely used broad-spectrum caspase inhibitor, Z-VAD-FMK (MedChemExpress), was used to treat Caco-2 cells and HIECs for 2 h, and the 0.5% DMSO solvent was used for comparison.

### Western blot assay

After treatment for 72 h, the cells were lysed and total proteins were extracted using RIPA buffer with protease inhibitors (Beyotime Biotechnology). Then, the protein concentration was determined by the BCA assay kit (Beyotime Biotechnology). Equal amounts of protein samples (40 μg) were separated by SDS-polyacrylamide gel electrophoresis and then transferred to PVDF membranes. The membranes were blocked in 5% fat-free milk, then cut according to the size of the target protein and incubated with primary antibodies against DLL4 (1:1000, Abcam), Notch (1:1000, Abcam), Hes1 (1:1000; Abcam), Bax (1:1000, Abways Technology), Bcl-2 (1:1000, Abways Technology), cleaved caspase-3 (1:1000, Cell Signaling Technology) and GAPDH (1:1000) (Beyotime Biotechnology) at 4 °C overnight and then incubated with secondary antibodies (1:4000, Beyotime Biotechnology) at room temperature for 1 h^39^. Finally, the bands were exposed by ECL (Tanon, Shanghai, China).

### Reporter vector construction and luciferase reporter assay

The conserved binding sequence of miR-30a-5p/miR-30e-5p in circRNA_103765 and its mutant sequence were cloned into the psiCHECK-2 luciferase reporter vector (GenScript), termed circRNA_103765-WT and circRNA_103765-Mut, respectively. The psiCHECK-2-circRNA_103765-WT (or circRNA_103765-Mut) reporter plasmid and miR-30a-5p/miR-30e-5p mimics (or negative control mimics) were cotransfected into 293T cells using Lipofectamine 3000 Transfection Reagent. The luciferase assay was performed by the Dual-Luciferase Reporter Assay System (Promega-E1910) according to the manufacturer’s protocols. The relative luciferase activity was expressed as the firefly to Renilla luciferase activity ratio.

### Fluorescence in situ hybridization (FISH)

The FISH assay was performed to observe the location of circRNA_103765 and miR-30a-5p/miR-30e-5p in Caco-2 cells and HIECs. Cells grown on coverslips were hybridized with a Cy3-labeled circRNA_103765 probe (Cy3-5′-GA + TGACCCTG CTTTG + TGCAT C + TGCATAGGT GT + TGGCCTTG-3′-Cy3) and FAM-labeled miR-30a-5p probe (FAM-5′-CTTCCAGTC + G + AGGATGTTTACA-3′-FAM) and miR-30e-5p probe (FAM-5′-CT + TCCAGTCA + AGGATGTT + TA CA-3′-FAM) (Genepharma). The slides were imaged with a confocal microscope (Leica/Zeisss).

### TNF-α measurement by flow cytometry (FCM)

The TNF­α level in plasma samples from patients with IBD was detected by FCM. The Cytometric bead arrays (CBA) (Hangzhou Cellgene Biotech Co., Ltd.) was applied to detect six cytokines (IL-2, IL-4, IL-6, IL-10, TNF-α and IFN-γ) simultaneously in the blood. According to the manufacturer’s instructions, The CBA technique was performed. The Dates were analyzed by the CellQuest Software 6.0 (BD Biosciences).

### Annotation of circRNA/miRNA interactions

MiRanda 2010 (http://www.microrna.org/) and TargetScan 7.2 (http://www.targetscan.org/) were applied to predict circRNA_103765/miRNA interactions. Additionally, the sequences of the MREs and predicted miRNA targets were analyzed and annotated in detail to facilitate our study.

### Ethics statement

All the studies were carried out in accordance with the approved guidelines. The study was approved by the Ethics Committees of the First Affiliated Hospital of Soochow University and the Affiliated Suzhou Hospital of Nanjing Medical University. The informed consent forms from all the participants were received.

### Statistical analysis

The results are presented as mean ± SD (standard deviation) or medians (1/4–3/4 quarters) and were obtained from three independent experiments. Normality (Kolmogorov–Smirnov) test was initially carried out and since the normality assumption was satisfied for the comparison of means between two groups, the paired *t* test or unpaired *t* test were used. For comparisons between multiple groups, one-way ANOVA was applied. For variables are skew distribution, Mann–Whitney's *U* test was applied. Spearman’s analysis was used to analyze the linear correlation between groups. The clinical diagnostic value of the candidate circRNA_103765 was assessed with a receiver operating characteristic (ROC) curve. A *P* value < 0.05 was considered statistically significant. All statistical analyses were performed using GraphPad Prism 7.04 (GraphPad Software, La Jolla, CA) and SPSS 19.0 (SPSS Inc., Chicago, IL, USA).

## Supplementary Information

Supplementary Information.

## Data Availability

All data generated or analyzed during this study are included in the manuscript.
