# Supplementary material for: CircRNA_103765 acts as a proinflammatory factor via sponging miR-30 family in Crohn’s disease
Source: Sci Rep. 2021 Jan 12;11:565. doi: 10.1038/s41598-020-80663-w (PMC7804428; doi:10.1038/s41598-020-80663-w)
Supplement: Supplementary file 1 — Supplementary Information. [file 41598_2020_80663_MOESM1_ESM.pdf]

CircRNA_103765 acts as a proinflammatory factor via sponging miR-30 family in Crohn's disease

Yulan Ye, Liping Zhang, Tong Hu, Juan Yin, Lijuan Xu, Zhi Pang,
Weichang Chen*

Supplementary Fig. S1

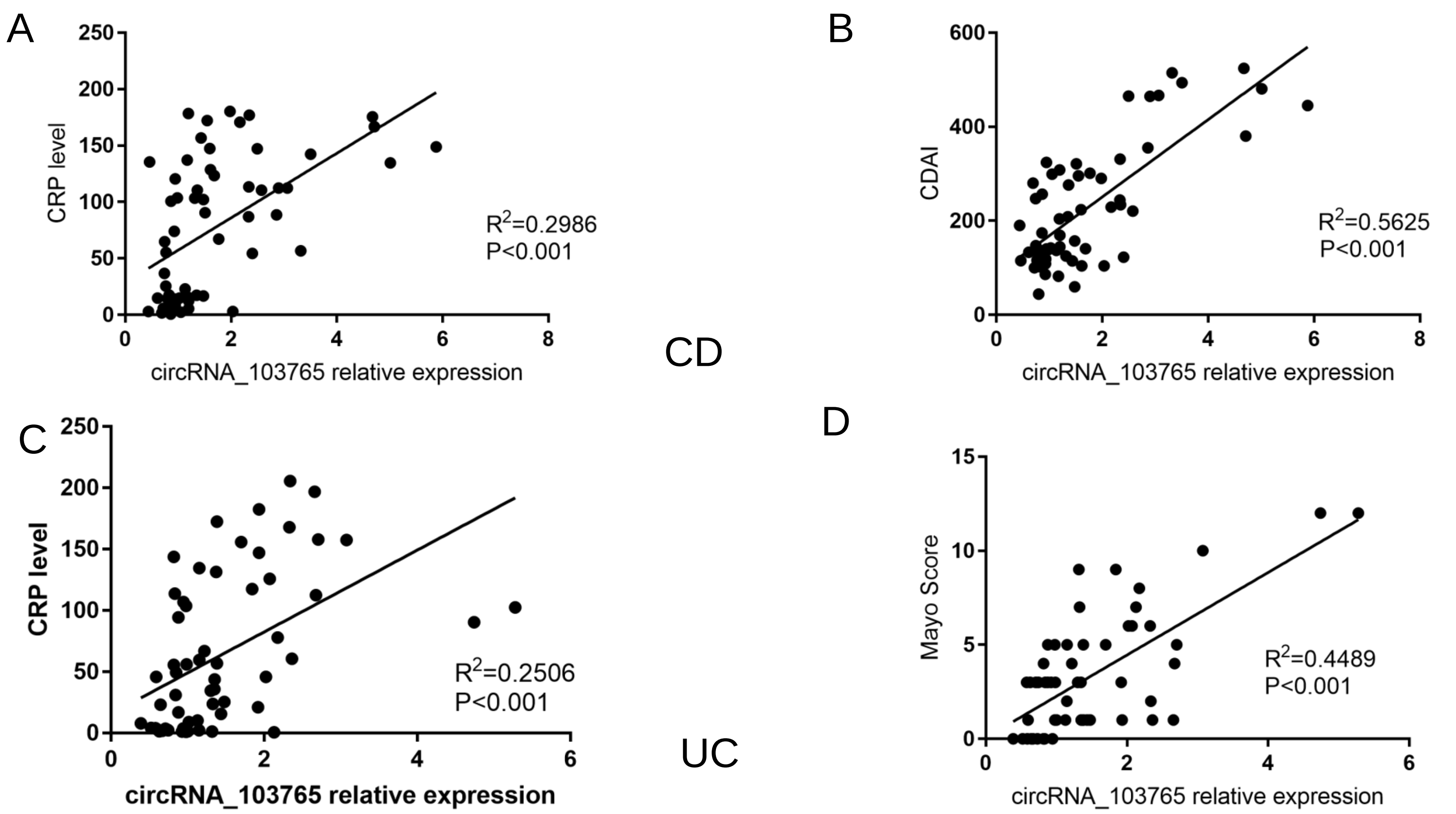

Supplementary Figure. S1 Correlations of circRNA_103765 expression with C reactive protein, Crohn's disease activity index, and Mayo score in inflammatory bowel disease patients. A and B Correlations of circRNA_103765 expression with disease activity in CD patients; C and D Correlations of circRNA_103765 expression with disease activity in UC patients.

Supplementary Fig.S2

miR-30a-5p

miR-30b-5p

miR-30d-5p

miR-30e-5p

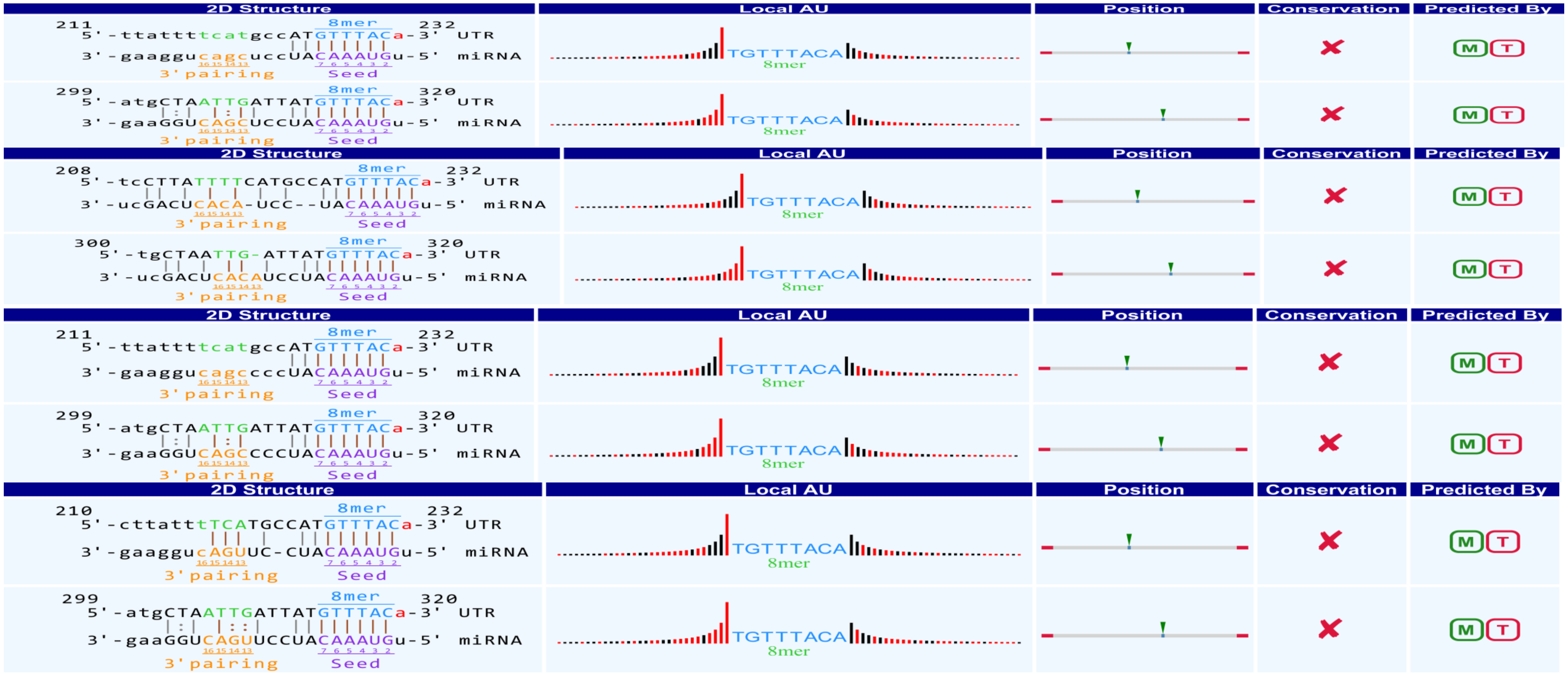

Supplementary Figure. S2 The miR-30 binding site on circRNA_103765 predicted by targetScan 7.2 (http://www.targetscan.org/) and miRanda 2010 (http://www.microrna.org/)

Supplementary Fig.S3

Before IFX

A

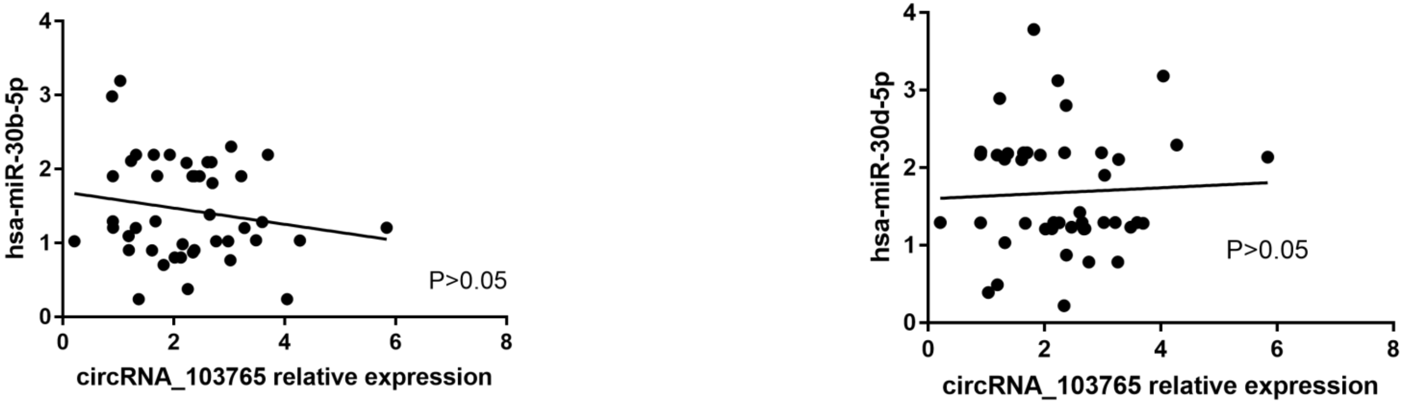

After IFX

B

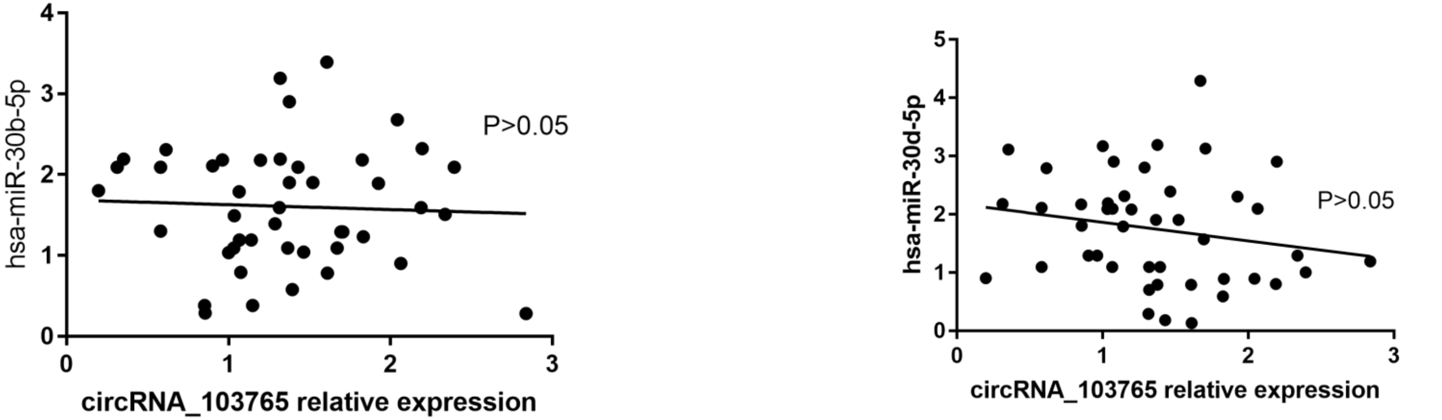

Supplementary Figure.S3 A and B Correlations of circRNA_103765 expression with miR-30b-5p and miR-30d-5p in active CD patients prior to and after IFX treatment (n=45).

Supplementary Table S1 Receiver-operating characteristic analysis of circRNA_103765 in peripheral blood mononuclear cells from patients with IBD and HCs

Group	AUC (95%CI)	*P*-value	Sensitivity (95%CI)	Specificity (95%CI)
CD *vs* HCs	0.7015(0.6263- 0.8066)	<0.001	53.33%(40.00%-66.33%)	77.50%(61.55%- 89.16%)
UC *vs* HCs	0.6526(0.5412- 0.7638)	0.01	80.00%(67.67%- 89.22%)	45.00%(29.26% - 61.51%)
Active CD *vs* HCs	0.7890(0.6957-0.8784)	<0.001	76.47%(58.83%- 89.25%)	70.00%(53.47% -83.44%)
Active UC *vs* HCs	0.7410(0.6070-0.8358)	0.001	88.57%(73.26%- 96.8%)	45.00%(29.26%- 61.51%)

CD: Crohn's disease; UC: Ulcerative colitis; HCs: Healthy controls; IBD: inflammatory bowel disease; AUC: Area under the curve; CI: confidence interval

Supplementary Table S2. Correlation coefficients between circRNA_103765 and clinical parameters in IBD patients

Variable	circRNA_103765	
	r	*P*
Active CD		
TNF-α	0.709	<0.001
CRP	0.483	0.003
CDAI	0.733	<0.001
Remission CD		
TNF-α	0.499	<0.001
CRP	0.361	0.057
CDAI	0.627	<0.001
Active UC		
TNF-α	0.714	<0.001
CRP	0.453	0.006
Mayo score	0.709	<0.001
Remission UC		
TNF-α	0.672	<0.001
CRP	0.313	0.061
Mayo score	0.225	0.278

CD: Crohn's disease; UC: Ulcerative colitis; IBD: inflammatory bowel disease; CRP: C-reactive protein; TNF-α: Tumor necrosis factor α; CDAI: CD Activity Index.

Figure3D

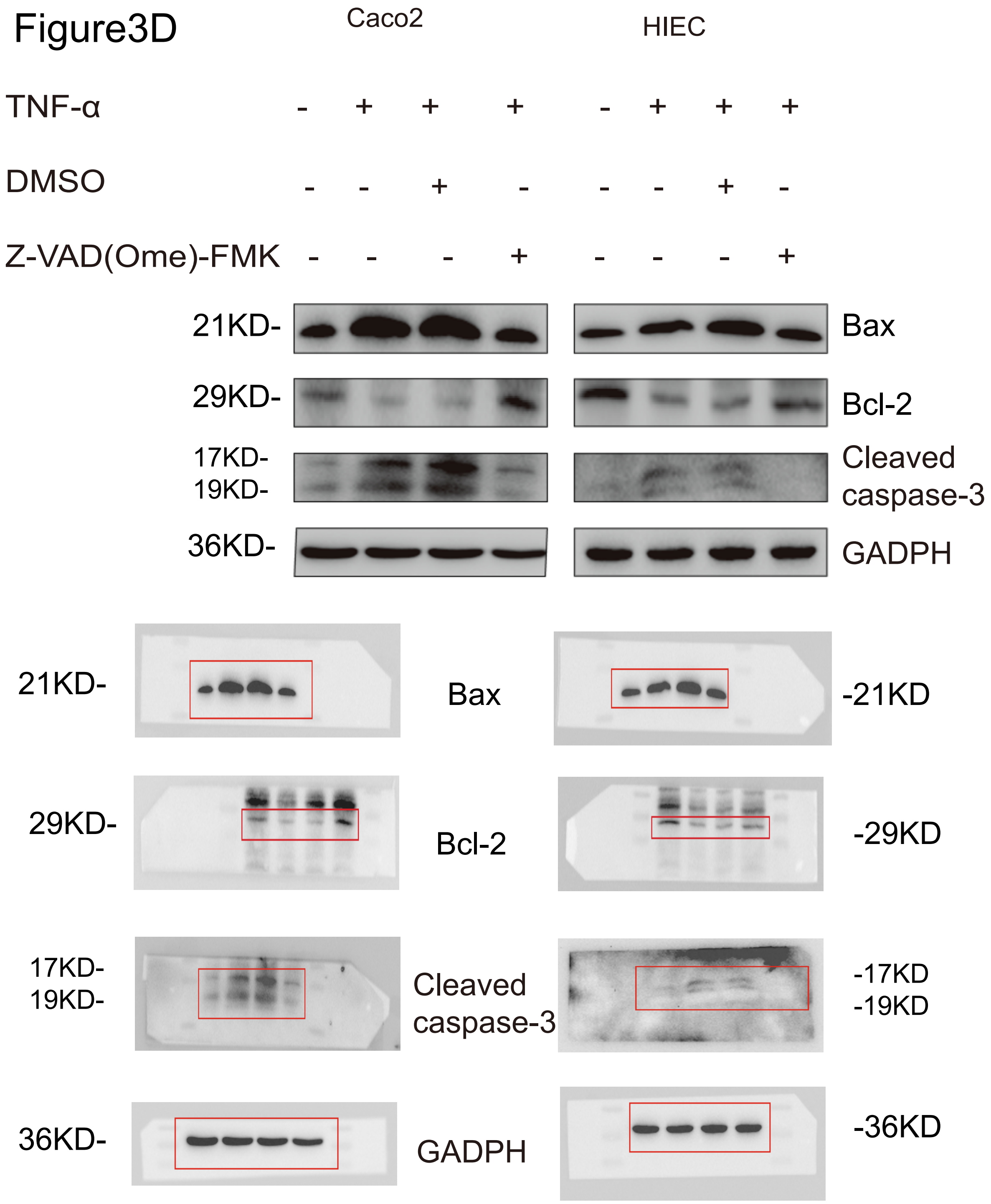
Caco2
HIEC
TNF-α
DMSO
Z-VAD(Ome)-FMK
21KD-
29KD-
17KD-
19KD-
36KD-
Bax
Bcl-2
Cleaved caspase-3
GADPH
-21KD
-29KD
-17KD
-19KD
-36KD

Figure4E

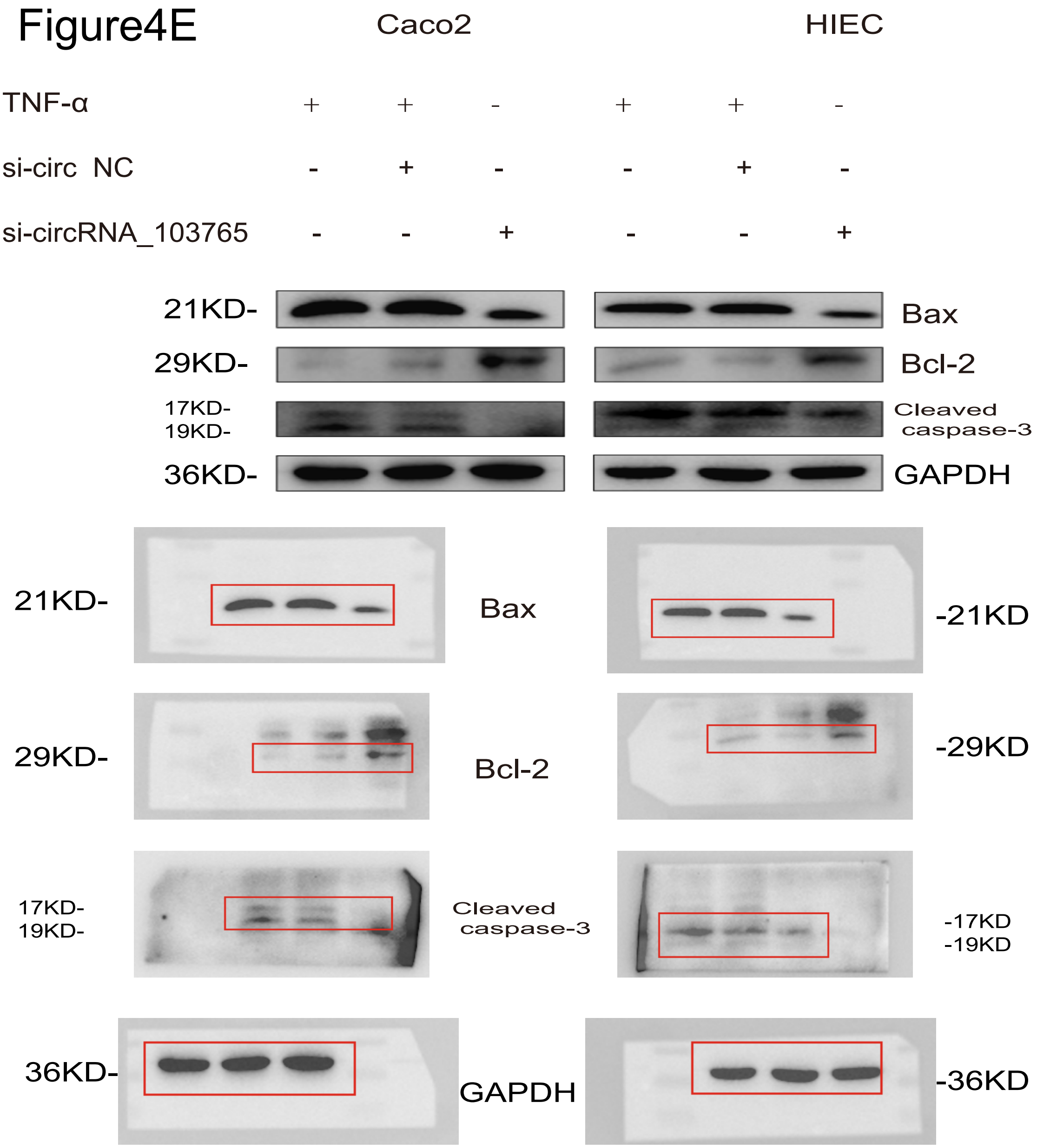
Caco2
HIEC
TNF-α
si-circ NC
si-circRNA_103765
21KD-
29KD-
17KD-
19KD-
36KD-
Bax
Bcl-2
Cleaved caspase-3
GAPDH
-21KD
-29KD
-17KD
-19KD
-36KD

Figure6G

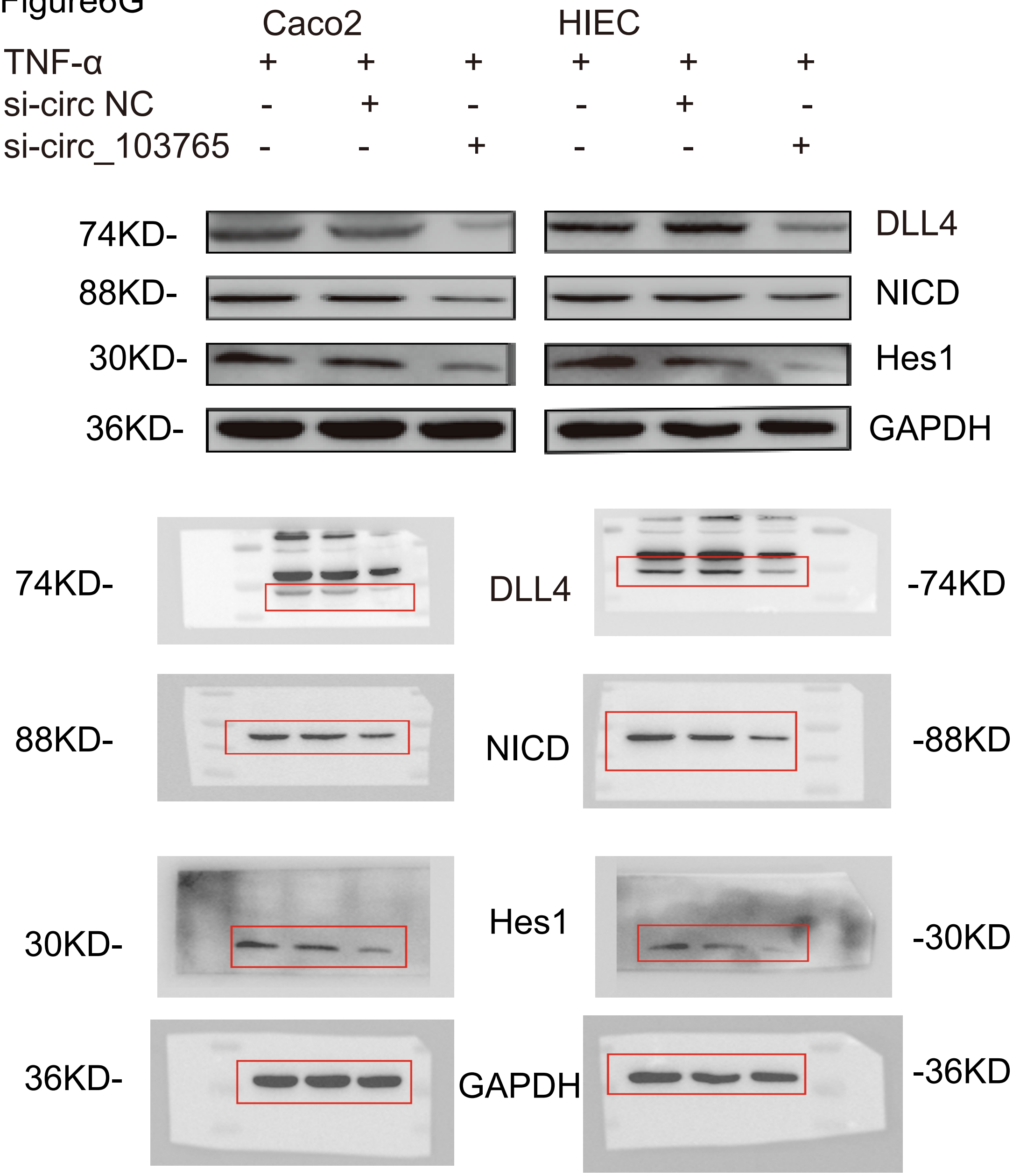
Caco2
HIEC
TNF-α + + + + + +
si-circ NC - + - - + -
si-circ_103765 - - + - - +
74KD-
DLL4
88KD-
NICD
30KD-
Hes1
36KD-
GAPDH
74KD-
DLL4
-74KD
88KD-
NICD
-88KD
30KD-
Hes1
-30KD
36KD-
GAPDH
-36KD

Figure6I

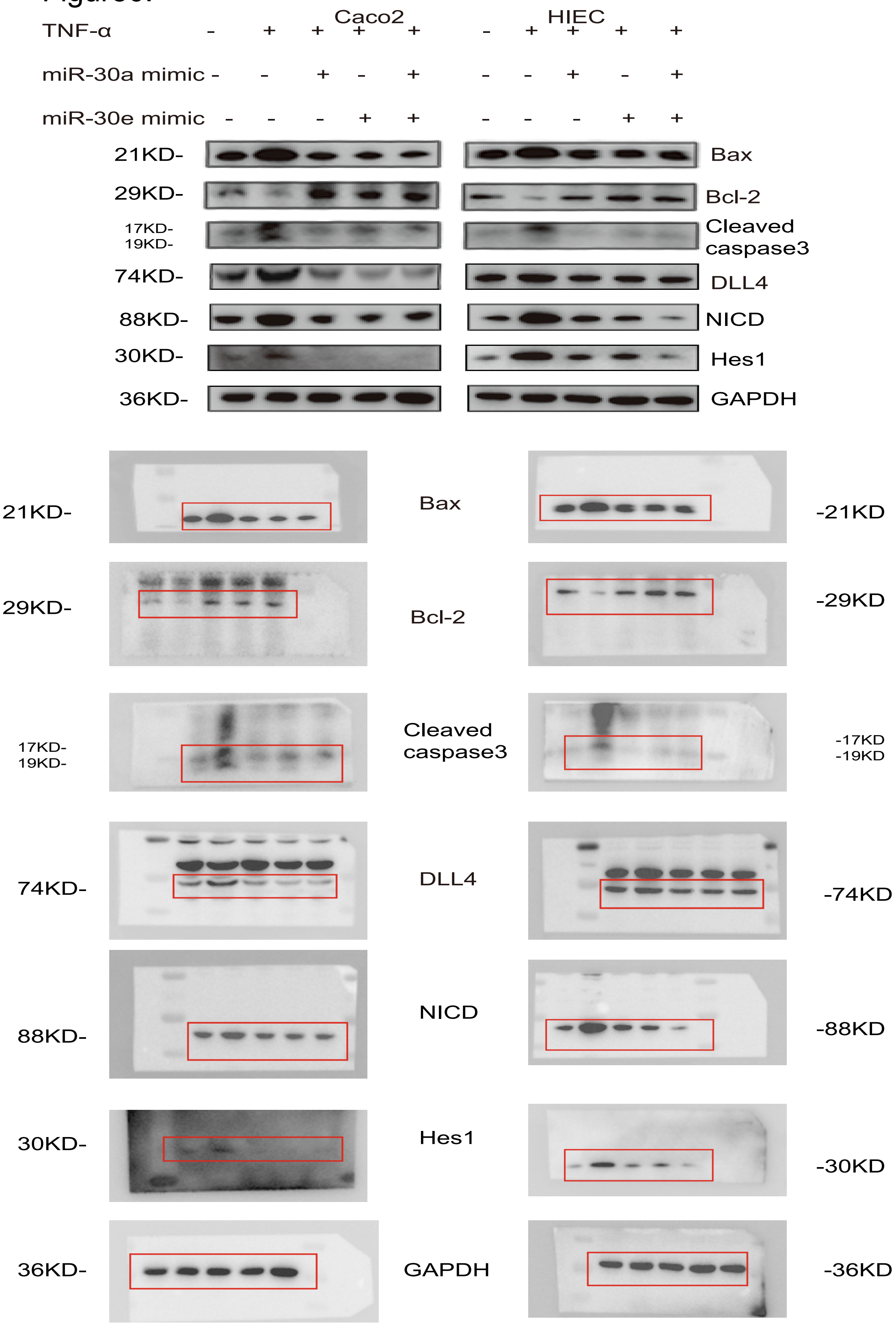
Caco2
HIEC
TNF-α - + + + + - + + + +
miR-30a mimic - - + - + - - + - +
miR-30e mimic - - - + + - - - + +
21KD-
Bax
29KD-
Bcl-2
17KD-
19KD-
Cleaved caspase3
74KD-
DLL4
88KD-
NICD
30KD-
Hes1
36KD-
GAPDH
21KD-
Bax
-21KD
29KD-
Bcl-2
-29KD
17KD-
19KD-
Cleaved caspase3
-17KD
-19KD
74KD-
DLL4
-74KD
88KD-
NICD
-88KD
30KD-
Hes1
-30KD
36KD-
GAPDH
-36KD

Figure6K

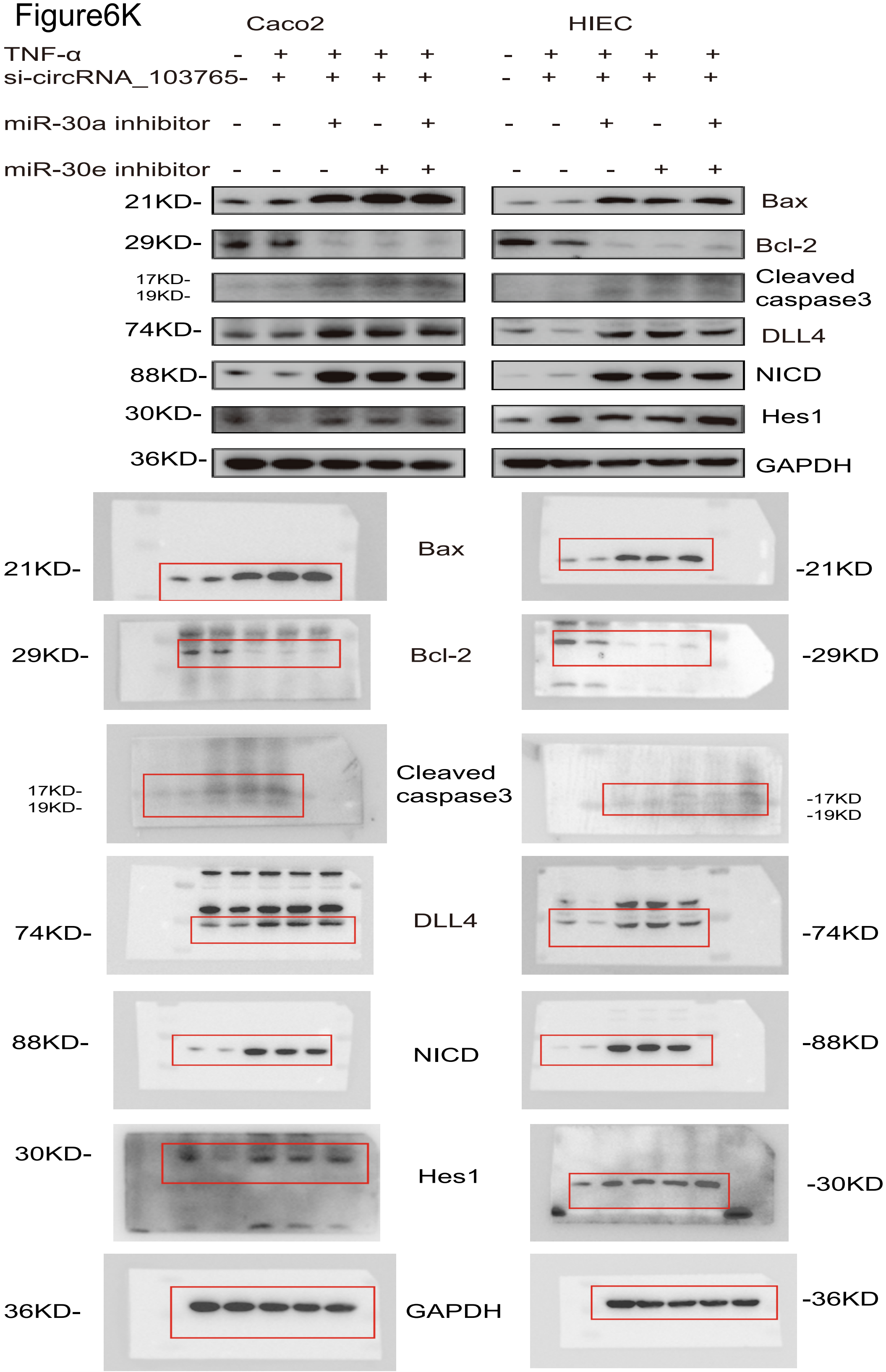

Caco2
HIEC
TNF-α
si-circRNA_103765-
miR-30a inhibitor
miR-30e inhibitor
21KD-
29KD-
17KD-
19KD-
74KD-
88KD-
30KD-
36KD-
Bax
Bcl-2
Cleaved caspase3
DLL4
NICD
Hes1
GAPDH
-21KD
-29KD
-17KD
-19KD
-74KD
-88KD
-30KD
-36KD
